# AN INTRODUCTION TO THE MAXIMIZING INDEPENDENCE AT HOME (MIND AT HOME) PROGRAM

**DOI:** 10.1093/geroni/igae098.1343

**Published:** 2024-12-31

**Authors:** Quincy Samus

**Affiliations:** Johns Hopkins University, Baltimore, Maryland, United States

## Abstract

People living with dementia (PLWD) represent some of the highest-need and highest-cost individuals living in the community. The complexity and range of needs (including medical, behavioral, and social needs) make dementia one of the most expensive chronic conditions in the U.S., and a strong candidate for improving care and reducing costs through care management support. The Maximizing Independence at Home (MIND at Home) dementia care program, developed and implemented in a variety of settings and contexts over the last 15 years, is a care coordination program for community-living persons with dementia and cognitive impairment and their informal caregiver. The MIND program consists of a comprehensive set of assessments, tools and staff trainings designed to increase the ability to stay at home in the community; reduce unmet care needs and improve quality of care; reduce caregiver burden and avoid high health care costs. The MIND at Home program model builds on three prior trials (two RCTs, one quasi-experimental design) of the MIND at Home dementia care coordination model and five practice-based implementations in real world practice settings to date. This session will review key components of the MIND at Home dementia care model, the characteristics of the care team, core training elements, and summarize research findings to date across the developmental pipeline.

